# Prolonged Active SARS-CoV-2 Infection in a Patient With Chronic Lymphocytic Leukemia on Venetoclax

**DOI:** 10.7759/cureus.116542

**Published:** 2026-09-19

**Authors:** Brian Laverentz, Nhu Do, Leslie A Jette-Kelly, Christina Schofield

**Affiliations:** 1 Internal Medicine, Madigan Army Medical Center, Tacoma, USA; 2 Pulmonology and Critical Care, Madigan Army Medical Center, Tacoma, USA; 3 Infectious Diseases, Madigan Army Medical Center, Tacoma, USA

**Keywords:** sars-cov-2 in malignancy, sars-cov-2 prolonged active infection, sars-cov-2 (severe acute respiratory syndrome coronavirus-2), sars-cov-2 testing, sars-cov-2 treatment

## Abstract

We report a case of prolonged active SARS-CoV-2 infection in a patient with chronic lymphocytic leukemia (CLL) receiving venetoclax. The patient demonstrated persistent symptoms over a span of 105 days and repeatedly positive SARS-CoV-2 polymerase chain reaction (PCR) testing. The case highlights the diagnostic and therapeutic challenges in immunocompromised hosts, including limited guidance and drug availability. Management required multiple antiviral regimens and consideration of alternative therapies. This case underscores the need for further research that can inform more robust guidelines and therapeutic strategies in this vulnerable population.

## Introduction

The emergence of SARS-CoV-2 and the resulting global pandemic, lasting from December of 2019 to May of 2023, has highlighted both the adaptability of the virus and the vulnerability of certain patient populations. Among these, individuals who are immunocompromised, such as those receiving chemotherapy, biologic therapies, or immunosuppressive agents [1], face particularly complex challenges in the diagnosis, treatment, and clearance of the virus. In these patients, viral persistence rather than a short-lived acute illness can result [1-4].

Prolonged or relapsing SARS-CoV-2 infection has been increasingly recognized in the literature [1-4] as a rare yet serious condition occurring in immunocompromised hosts. While no universally accepted definition currently exists, persistent SARS-CoV-2 infection has been broadly characterized as continued polymerase chain reaction (PCR) positivity with compatible symptoms lasting beyond 21 to 30 days in the setting of significant immunosuppression [3]. These cases often lack the typical immune-assisted viral clearance [2,5,6], resulting in extended periods of viral shedding and potential for progressive disease. The clinical approach to such patients is further complicated by the paucity of evidence-based guidelines, the evolving landscape of antiviral and monoclonal antibody therapies, and the consideration of drug interactions and immune modulation [4].

Chronic lymphocytic leukemia (CLL) is an indolent B-cell lymphoproliferative malignancy associated with well-characterized immune dysfunction affecting both humoral and cellular compartments. The malignant B-cell clone disrupts normal immune architecture, contributing to hypogammaglobulinemia and impaired T-cell function that collectively reduce the host's capacity for effective antiviral response [5]. Venetoclax, a selective BCL-2 inhibitor used in the treatment of CLL, further compounds this immune deficit by promoting apoptosis of BCL-2-overexpressing B cells, thereby impairing the generation of antigen-specific antibody responses necessary for viral clearance [4,5]. The convergence of these two mechanisms, CLL-associated immune dysregulation and venetoclax-mediated B-cell suppression, creates a permissive environment for prolonged viral persistence, providing distinction in this patient population from other immunocompromised hosts.

This report discusses the prolonged course of SARS-CoV-2 in a patient with CLL receiving venetoclax, a BCL-2 inhibitor, and the evolving therapeutic decisions and clinical monitoring strategy utilized during their 105-day illness. By presenting the complex clinical decision-making and evolving response to treatment alongside the immunological context underlying this patient's vulnerability, we aim to contribute to the growing understanding of SARS-CoV-2 in immunocompromised patients.

## Case presentation

A 69-year-old male with active CLL and 11q deletion, diagnosed over three years prior, on a stable dose of venetoclax, presented initially in January 2024, approximately six months after the end of the SARS-CoV-2 pandemic, with a productive cough and sore throat. He had completed the primary SARS-CoV-2 mRNA vaccination series (Pfizer Inc., New York, NY, USA, and BioNTech SE, Mainz, Germany) with three additional boosters. At initial presentation (Day 0), he was hemodynamically stable with a SpO₂ of 99% on room air and a mildly reduced tympanic temperature of 35.8°C (Table 1, Figure 1). He was diagnosed with SARS-CoV-2 via antigen testing. A confirmatory nucleic acid amplification test was not performed at the time of initial diagnosis, and no cycle threshold (Ct) value was therefore obtainable at disease onset.

**Table 1:** Patient's vital signs at the time of presentation. Demonstrating hemodynamic stability and a mildly reduced temperature not meeting the criteria for hypothermia (<35 degrees C). C: Celsius

Vital Signs	Measurement	Units
Blood Pressure	133 / 67	mmHg
Heart Rate	68	per minute
Respiratory Rate	18	per minute
SpO_2_	99	%
Tympanic Temperature	35.8	C

**Figure 1:**
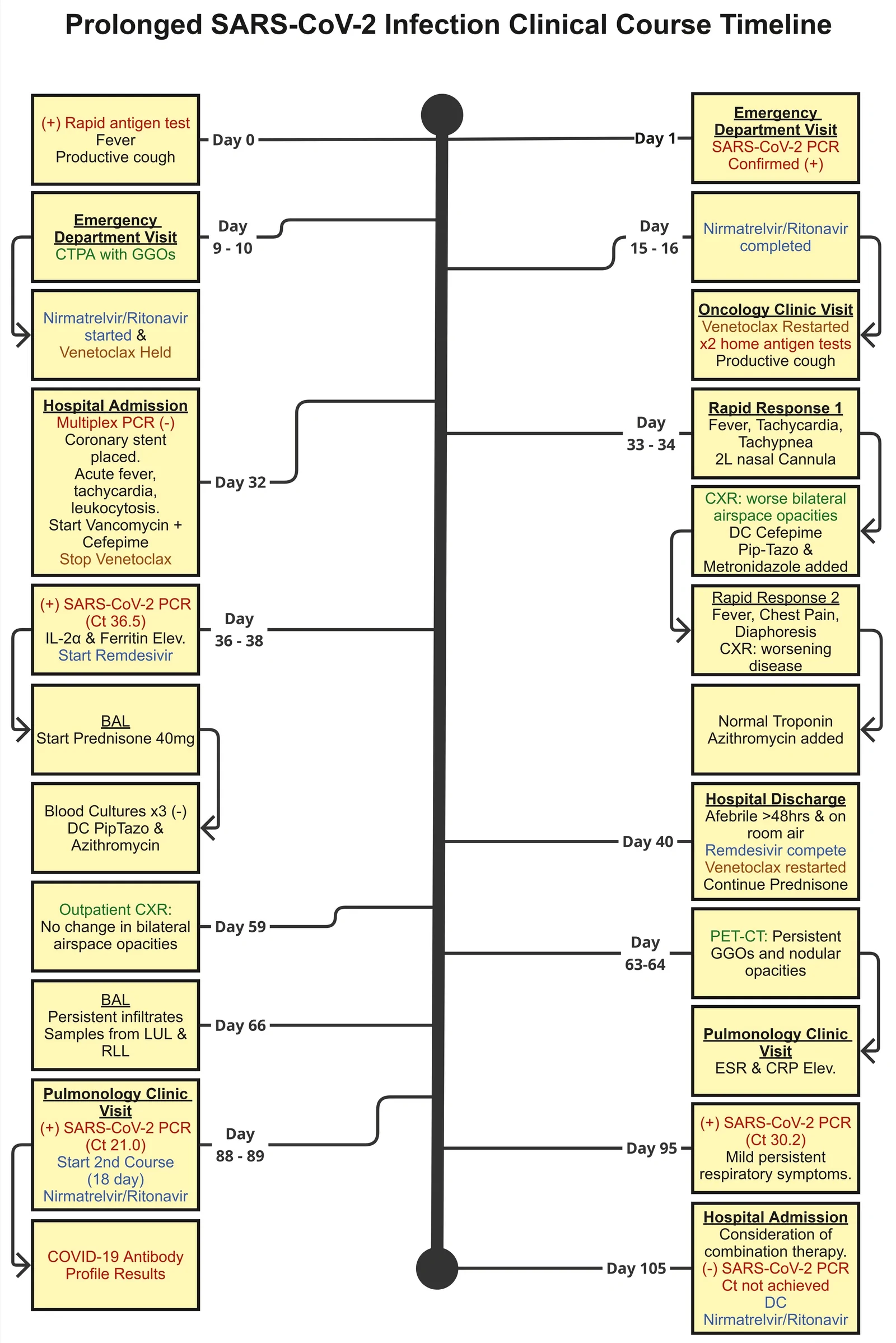
Detailed timeline from first positive SARS-CoV-2 test covering emergency department visits, hospital admissions, clinic visits, medication changes, imaging, pertinent temporal labs, and SARS-CoV-2 testing with final negative result. Red text: viral testing; blue text: antiviral therapies; brown text: chemotherapeutics; green text: radiographic imaging. GGOs: ground-glass opacities; CXR: chest X-ray; Ct: cycle threshold; IL-2α: interleukin 2-alpha; elev.: elevated/elevation; (+): positive result; (-): negative result; PET-CT: positron emission tomography- computed tomography; BAL: bronchoalveolar lavage; ESR: erythrocyte sedimentation rate; CRP: C-reactive protein; CTPA: computed tomography pulmonary angiography; LUL: left upper lobe; RLL: right lower lobe; DC: discontinue(d) Image credits: Authors, using Miro (RealtimeBoard, Inc.), San Francisco, CA, USA.

He completed a five-day course of nirmatrelvir/ritonavir (Paxlovid) beginning Day 10, with temporary cessation of venetoclax due to the CYP3A4 inhibitory effect of ritonavir and concern for increased venetoclax plasma exposure. Venetoclax was restarted on Day 16 at an oncology follow-up visit, the day after completion of the antiviral course. On Day 9, he presented to the emergency department with shortness of breath and underwent CT pulmonary angiography, which demonstrated multifocal multilobar ground-glass opacities without pulmonary embolism (Video 1). At that visit, laboratory evaluation revealed a mildly decreased white blood cell count, a normal absolute neutrophil count, and a low absolute lymphocyte count reflecting venetoclax-mediated B-cell suppression. Hepatic enzymes were mildly elevated, representing values present at baseline prior to this illness. Procalcitonin was 0.28 ng/mL (reference ≤0.5 ng/mL), arguing against a primary bacterial process (Table 2).

**Video 1:** Emergency department computed tomography with pulmonary angiography. Demonstrating the absence of pulmonary embolism and the presence of multifocal multilobar ground-glass opacities consistent with SARS-CoV-2 pulmonary infection, in addition to mediastinal and systemic lymphadenopathy compatible with a history of lymphoma.

**Table 2:** Relevant lab testing that occurred over the entire clinical course of the case. Trends notable and supportive of SARS-CoV-2 infection include (A) white blood cell (WBC) counts that show an initial mild leukopenia with a rise and subsequent fall with resolution of infection, never exceeding the upper limit of normal. (B) Absolute neutrophil and (C) absolute lymphocyte counts remain within normal limits despite active infection, consistent with immunosuppression. (D) Liver enzymes showed initial mild elevations with resolution toward the end of the clinical course, and (E) inflammatory markers support the active inflammatory process of infection. (F) Procalcitonin at the initial emergency department visit indicated a lower likelihood of bacterial infection at that time. On dates in which (G) cycle thresholds were obtained with PCR testing, they were also noted and showed no consistent trend; however, they are indicative of COVID positivity and support the presence of infection without signs of increased viral burden rather than improvement before the final testing, at which point the cycle threshold was not achieved to indicate the presence of infection. WBC: white blood cell; uL: microliter; Ref: reference range; Abs Neut: absolute neutrophils; Abs Lymph: absolute lymphocytes; Alk Phos: alkaline Phosphatase; U/L: units per liter; ALT: alanine aminotransferase; AST: aspartate aminotransferase; ESR: erythrocyte sedimentation rate; mm/hr: millimeters per hour; CRP: C-reactive protein; mg/dL: milligrams per deciliter; Procal: procalcitonin; PCR: polymerase chain reaction

Day	^A^WBC 10^3/uL (Ref. 4.5-13)	^B^Abs Neut 10^3/uL (Ref. 1.5-10)	^C^Abs Lymph 10^3/uL (Ref 0.9-3.0)	^D^Alk Phos U/L (Ref 35-147)	^D^ALT U/L (Ref 7-42)	^D^AST U/L (Ref 8-48)	^E^ESR mm/hr (Ref. <20 mm/hr)	^E^CRP mg/dL (Ref. <1)	^F^Procal ng/mL (Ref ≤0.5)	^G^SAR-CoV-2 RT-PCR Detection cycles (Ref. none)
9	4.3	3.43	0.21	150	66	69			0.28	
32	8.1	6.03	0.45							
36										36.5
37								33		
40	7.8	6.28	0.46							
64	8.8	7.35	0.94				120	23.2		
88										21
95										30.2
105	6.1	5.02	0.69	145	17	13	20	1.9		0 (undetected)
106	5.7	3.95	0.79							

Following completion of the antiviral course, the patient reported two negative home rapid antigen tests within the two days prior to Day 18 and overall improvement in symptoms, though residual cough with white phlegm persisted. 

On Day 32, the patient underwent elective percutaneous coronary intervention (PCI) for a high-grade mid-left anterior descending artery stenosis. Post-procedurally, he developed fever, tachycardia, and leukocytosis. A multiplex respiratory panel (BioFire Diagnostics, LLC, Salt Lake City, UT, USA) performed at admission on Day 32 returned pan-negative (testing for SARS-CoV-2, influenza A & B, respiratory syncytial virus, human metapneumovirus, human rhinovirus & enterovirus, adenovirus, parainfluenza viruses 1 through 4, corona virus subtypes, *Bordetella pertussis, Chlamydia pneumoniae*, and *Mycoplasma pneumoniae*); however, given the clinical picture and the patient's immunocompromised status, Infectious Disease was consulted, and a dedicated SARS-CoV-2 PCR with Ct reporting was ordered on Day 36, returning positive with a Ct of 36.5 (Table 2).

Laboratory evaluation during this admission demonstrated an increase in white blood cell count, absolute neutrophil count, and absolute lymphocyte count from previous testing without exceeding upper limits of the reference range. Further testing with C-reactive protein (CRP) and ferritin (3,761 ng/mL; reference 30-400 ng/mL) demonstrated significant marked elevations, with a procalcitonin level that was unremarkable (Table 2). Interleukin-2 receptor alpha was also assessed, demonstrating significant elevations as well at 1,415 U/mL (reference 30-400 U/mL), indicative of significant immune activation. Initial fungal workup was unremarkable, with beta-D-glucan <31.25 pg/mL (reference <60) and *Coccidioides* antibody negative.

Given significant concerns regarding the patient's immune compromise and cancer history, he was treated empirically during that admission for bacterial pneumonia with vancomycin, cefepime, and later metronidazole given concerns regarding response to antibiotics. These antibiotics were later exchanged for piperacillin-tazobactam and azithromycin for broader coverage as a result of persistent fever, then discontinued on Day 38 as blood cultures from Days 32, 34, and 36 showed no growth and the methicillin-resistant *Staphylococcus aureus *(MRSA) nares screen was negative. He received a five-day course of intravenous remdesivir (Days 36-40) per infectious disease recommendations, and prednisone 40 mg daily was initiated on Day 37 at the recommendation of the pulmonary consultant. Bronchoscopy with bronchoalveolar lavage performed on Day 37 demonstrated no significant fungal or bacterial growth, negative *Pneumocystis* PCR, negative acid-fast bacilli smear and culture, and negative galactomannan, which at that time supported a post-COVID inflammatory etiology consistent with cryptogenic organizing pneumonia (COP). Venetoclax was held throughout this admission. During the hospital course, the patient required supplemental oxygen via nasal cannula in the setting of fever and worsening respiratory status, with documented oxygen saturation of 94% on 2 liters via nasal cannula during an overnight rapid response event on Day 34. He was subsequently weaned and discharged on Day 40 on room air. Venetoclax was restarted on discharge with ongoing prednisone 40 mg daily.

Following discharge, cough persisted beyond two weeks. A chest X-ray on Day 59 demonstrated no significant change in bilateral airspace opacities, consistent with organizing pneumonia (Figure 2). PET-CT on Day 63 demonstrated persistent multifocal ground-glass and nodular opacities interpreted as evolving multifocal pneumonia or other inflammatory etiology, with the caveat that superimposed pulmonary involvement of the known lymphoproliferative disease could not be excluded (Video 2).

**Figure 2:**
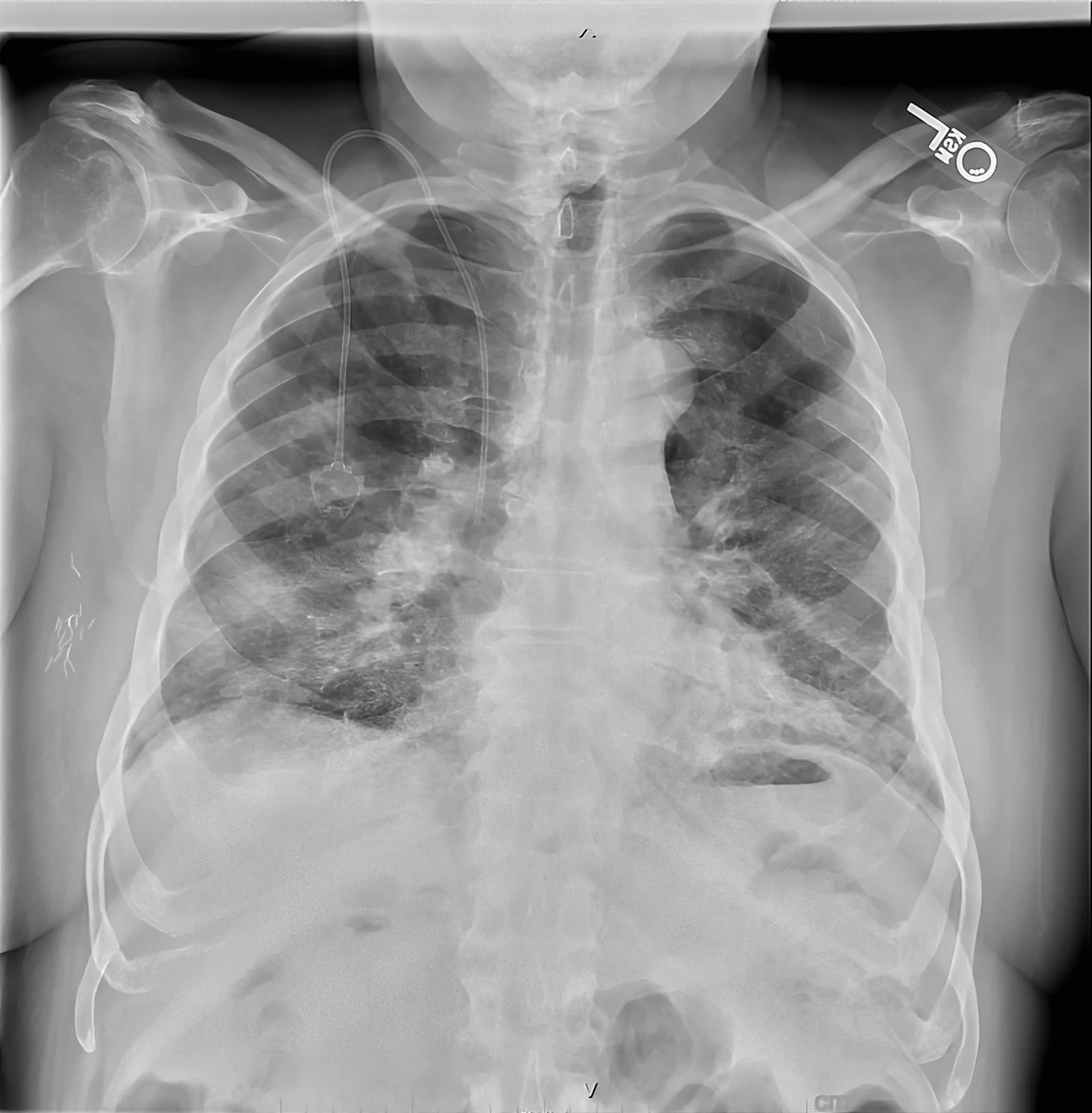
Chest radiograph performed on Day 59. Showing no significant change in bilateral airspace opacities and consistent with a history of organizing pneumonia.

**Video 2:** Computed tomography as part of positron emission tomography on Day 63. Demonstrating persistent multifocal ground-glass and nodular opacities, the radiologist also noted that superimposed pulmonary involvement of known lymphoproliferative disease could not be excluded.

At pulmonology follow-up on Day 64, erythrocyte sedimentation rate (ESR) was found to be elevated, and CRP remained elevated (Table 2). In combination with the PET CT findings (Video 2), consideration of prolonged active SARS-CoV-2 infection was formally noted.

COVID-19 antibody profile testing performed on Day 89 demonstrated a negative nucleocapsid antibody, positive spike antibody with a semi-quantitative spike antibody level of 7.9 (reference: negative if <0.8). While the clinical context for this testing did not allow for definitive attribution to vaccination versus prior infection-induced immunity, the absence of nucleocapsid antibody and the relatively low magnitude of the spike antibody response was viewed as most consistent with the impairment of humoral immunity seen in patients with CLL receiving venetoclax despite prior vaccination.

Serial SARS-CoV-2 PCR testing demonstrated continued positivity, with a Ct of 21.0 on Day 88 (Table 2), and a second 18-day course of nirmatrelvir/ritonavir was initiated, with venetoclax again held. Repeat PCR on Day 95 remained positive with a Ct of 30.2 (Table 2). At completion of the course, persistent PCR positivity with ongoing mild respiratory symptoms prompted consideration of combination antiviral therapy and high-titer convalescent plasma. The patient was admitted on Day 105 for intravenous antiviral therapy. Antibiotics were not initiated given the absence of clinical or laboratory evidence of bacterial superinfection, with the admission directed specifically at antiviral treatment of persistent SARS-CoV-2. PCR performed at the time of admission returned negative with with a Ct of 0, indicating the amplification threshold was not reached (Table 2), and CT chest demonstrated significant interval improvement in multifocal airspace disease. The patient was discharged on Day 106 with a prednisone taper and plans for venetoclax resumption following hematology-oncology follow-up.

## Discussion

Prolonged SARS-CoV-2 infection is a rare and challenging entity, typically limited to severely immunocompromised patients. In these cases, viral clearance may be significantly delayed due to an impaired ability to mount an effective immune response. As a result, these patients may experience a relapsing or persistent clinical course marked by intermittent symptom flares and ongoing radiographic abnormalities [1,2,4,7,8]. It should be noted, however, that there is no universally accepted definition and timeline for what constitutes a prolonged active SARS-CoV-2 infection at this time, and standardization may be helpful in future research on the topic.

Treatment strategies remain unstandardized, though emerging data support the use of extended course or combination antiviral therapy and monoclonal antibodies when available with favorable outcomes [3,5,8-11]. In the case presented, the patient required multiple rounds of antiviral therapy, including an extended course, and a prolonged clinical observation period. While high-titer convalescent plasma was considered [2] due to the inaccessibility of monoclonal antibodies and clinical improvement, this was not required.

Ct values from PCR testing [3] were also used to help guide clinical decision-making and assess for active viral replication. While not routinely used in immunocompetent individuals, lower Ct values may indicate higher viral loads and active infection, especially in symptomatic immunocompromised patients. In this case, a Ct value of 30.2 with continued symptoms suggested ongoing active viral presence and supported the decision to continue therapy. Although Ct is not recommended for therapeutic monitoring [3], it may provide additional context in prolonged infections where symptom resolution, imaging, and virology may not agree.

Prolonged SARS-CoV-2 infection is typically limited to severely immunocompromised patients. Treatment strategies remain unstandardized, though current available literature, composed of case reports and expert consensus, overall supports the use of dual antiviral therapy [8] and monoclonal antibodies [6] when available [3,4,10]. Due to limited access to monoclonals, convalescent plasma was considered in this case. Convalescent plasma would have served as an alternative to monoclonal antibodies given the concept of antibodies against the virus being present [6,7,11,12].

Several limitations of this report warrant acknowledgment. Definitive distinction between persistent infection and reinfection cannot be established, as viral genomic sequencing, SARS-CoV-2 culture, and subgenomic RNA assays were not performed due in part to resource limitations. This challenge is further compounded by the absence of a universally accepted definition of prolonged active SARS-CoV-2 infection, meaning no formal diagnostic criteria exist against which this case can be adjudicated. The argument for persistence over reinfection rests on a convergence of laboratory, radiographic, and clinical findings, most notably among these being continuously unresolved respiratory symptoms, a consistent and progressing radiographic pattern, clinical deterioration temporally linked to procedural stress, laboratory values suggesting subclinical immune activation in the interval period, and attenuated humoral immunity evidenced by a low-magnitude spike antibody response despite full vaccination. Individually, none of these findings is definitive, but collectively they are believed to support this interpretation. However, even in the case of reinfection after cardiac catheterization, total illness duration would exceed 90 days, representing a significantly prolonged course of illness. Additional limitations include the absence of a Ct value at initial diagnosis, the inability of the multiplex respiratory panel used on Day 32 to report Cts, the unavailability of the antigen assay manufacturer and home test brand, and the lack of immunoglobulin level measurement during the clinical course. 

Despite the limitations, this case adequately emphasizes the need for individualized care and flexibility in therapeutic planning, particularly with respect to drug-drug interactions such as the CYP3A4 inhibition exhibited by ritonavir, which increases venetoclax plasma exposure and necessitated repeated interruption of CLL-directed therapy [4]. The balance between managing immunosuppression and facilitating viral clearance further highlights the importance of interdisciplinary coordination in such cases. 

## Conclusions

This case underscores three core learning points for clinicians managing SARS-CoV-2 in patients with hematologic malignancies. First, the combination of CLL-associated immune dysfunction and venetoclax-mediated B-cell suppression represents a uniquely high-risk for prolonged viral persistence, warranting heightened vigilance even when initial clinical improvement is observed. Second, the CYP3A4 inhibitory properties of ritonavir necessitate repeated interruption of venetoclax during nirmatrelvir/ritonavir courses, requiring proactive interdisciplinary coordination. Third, the absence of evidence-based guidelines for this population means management must be individualized, iterative, and informed by the best available evidence and resources at a given institution while acknowledging its limitations. Prospective research and standardized diagnostic frameworks for prolonged SARS-CoV-2 infection in immunocompromised patients remain an urgent need.
